# Crosstalk Among YAP, LncRNA, and Tumor-Associated Macrophages in Tumorigenesis Development

**DOI:** 10.3389/fonc.2021.810893

**Published:** 2022-01-06

**Authors:** Jing Xu, Xin-Yuan Liu, Qi Zhang, Hua Liu, Peng Zhang, Zi-Bin Tian, Cui-Ping Zhang, Xiao-Yu Li

**Affiliations:** ^1^ Department of Gastroenterology, The Affiliated Hospital of Qingdao University, Qingdao, China; ^2^ Innovation Platform of Marine Drug Screening & Evaluation, Qingdao Pilot National Laboratory for Marine Science and Technology, Qingdao, China

**Keywords:** lncRNA, TAMs, YAP, tumorigenesis, Hippo pathway

## Abstract

Long non-coding RNAs (ncRNAs), which do not encode proteins, regulate cell proliferation, tumor angiogenesis, and metastasis and are closely associated with the development, progression, and metastasis of many cancers. Tumor-associated macrophages (TAMs) in the tumor microenvironment play an important role in cancer progression. The Hippo signaling pathway regulates cell proliferation and apoptosis, maintains tissue and organ size, and homeostasis of the internal environment of organisms. Abnormal expression of Yes-associated protein (YAP), the Hippo signaling pathway key component, is widely observed in various malignancies. Further, TAM, lncRNA, and YAP are currently valuable targets for cancer immunotherapy. In this review, we have logically summarized recent studies, clarified the close association between the three factors and tumorigenesis, and analyzed the outlook of tumor immunotherapy.

## Introduction

The human genome, which typically encodes both coding and non-coding transcripts, contains a large amount of apparently functional but non-coding DNA that is much larger than coding RNAs and estimated to be at least four times larger than protein-coding sequences (1). Long noncoding RNAs (lncRNAs) are endogenous nonprotein-coding RNAs larger than 200 nt that regulate biological processes such as tumor growth, proliferation, invasion, and metastasis at the epigenetic, transcriptional, or post-transcriptional level (2).

**Figure 1:**
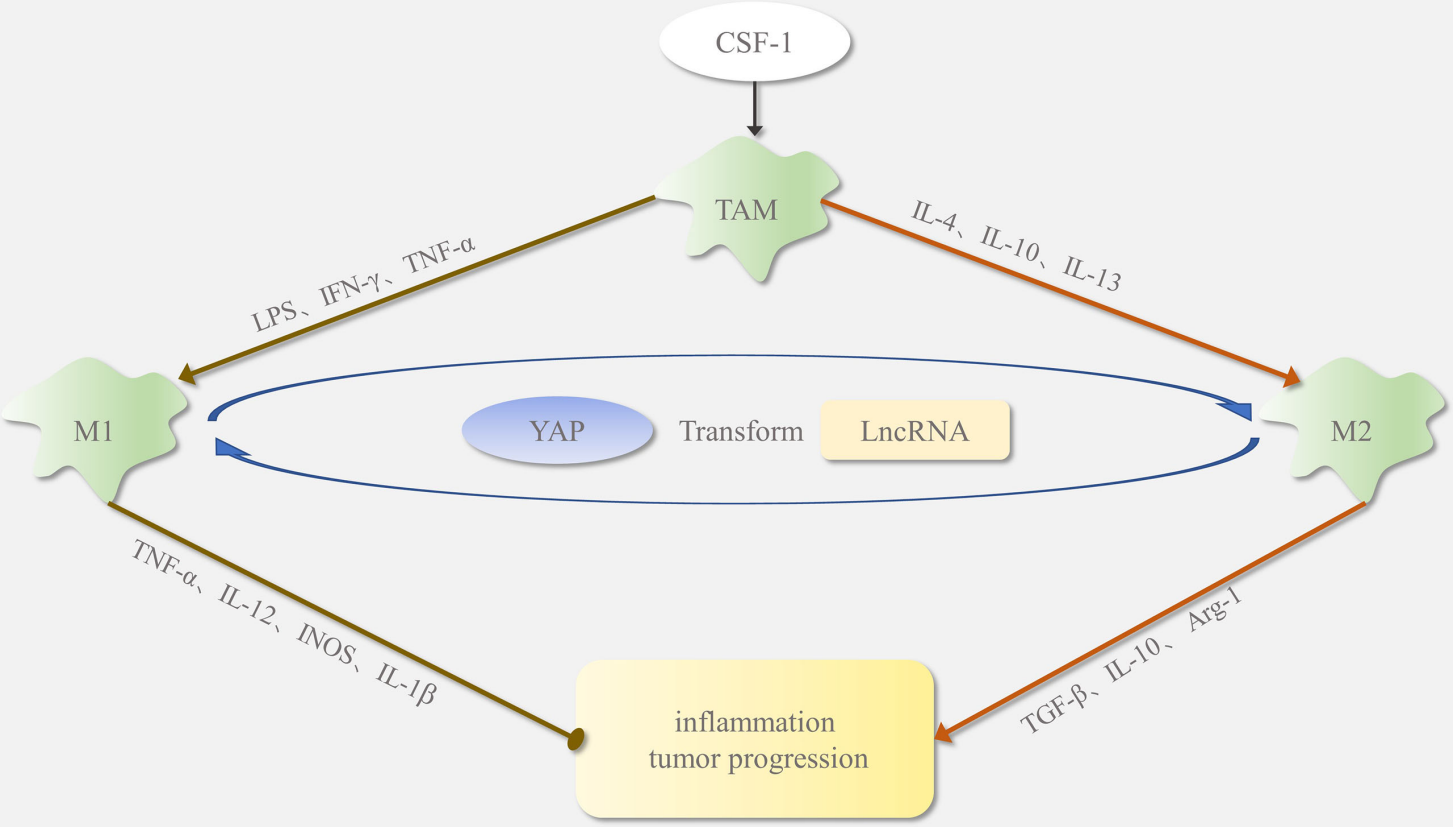
Polarization of tumor-associated macrophages (TAMs): In the tumor microenvironment, macrophages are usually recruited by CSF1. In response to different signaling stimuli, macrophages polarize into M1 and M2 phenotypes. Factors such as LPS and IFN-γ regulate TAM polarization toward the M1 type. The M1-phenotype macrophages secrete INOS, IL-1β, and TNF-α as proinflammatory stimuli, which promote inflammatory responses and inhibit tumor growth. Factors such as IL-4 and IL-13 regulate TAM polarization toward the M2 type. The M2-phenotype macrophages release TGF-β, Arg-1, and IL-10, which inhibit the inflammatory response and promote tumor formation. Yap protein and lncRNA affect M1/M2 phenotypic transition.

Tumor-associated macrophages (TAMs) in the tumor microenvironment (TME) are a major infiltrating non-cancerous cells and are closely associated with tumor proliferation, metastasis, invasion, and immune escape (3). LncRNAs have a regulatory relationship with TAMs during tumor development, and both have emerged as new therapeutic targets for cancer. TAMs are classified into M1 and M2 phenotypes in different tissue environments (4, 5). As shown in **Figure 1**, under the proinflammatory and antitumor conditions induced by lipopolysaccharide (LPS) and tumor necrosis factor-α (TNF-α), TAMs differentiate to the M1 phenotype (6); however, with the anti-inflammatory and protumorigenic effects induced by IL-10 and IL-4, TAMs polarize towards the M2 phenotype. Most macrophages exhibit the M1 phenotype early and inhibit tumor growth. In contrast, as tumor progression continues, macrophages gradually converge to the M2 phenotype, promoting tumor development (7). Increasing evidence suggests that the induction of macrophages from an M1 to M2 phenotype might promote cancer initiation and progression by inducing cell proliferation, metastasis, drug resistance, and immune evasion. The regulatory mechanisms of macrophage polarization are not clear, but lncRNA and Yes-associated protein (YAP) proteins play a significant role in the M1/M2 switch. An in-depth study of the mechanisms mediating TAM phenotypic transition will provide a new direction for the treatment of malignancies.

**Figure 2:**
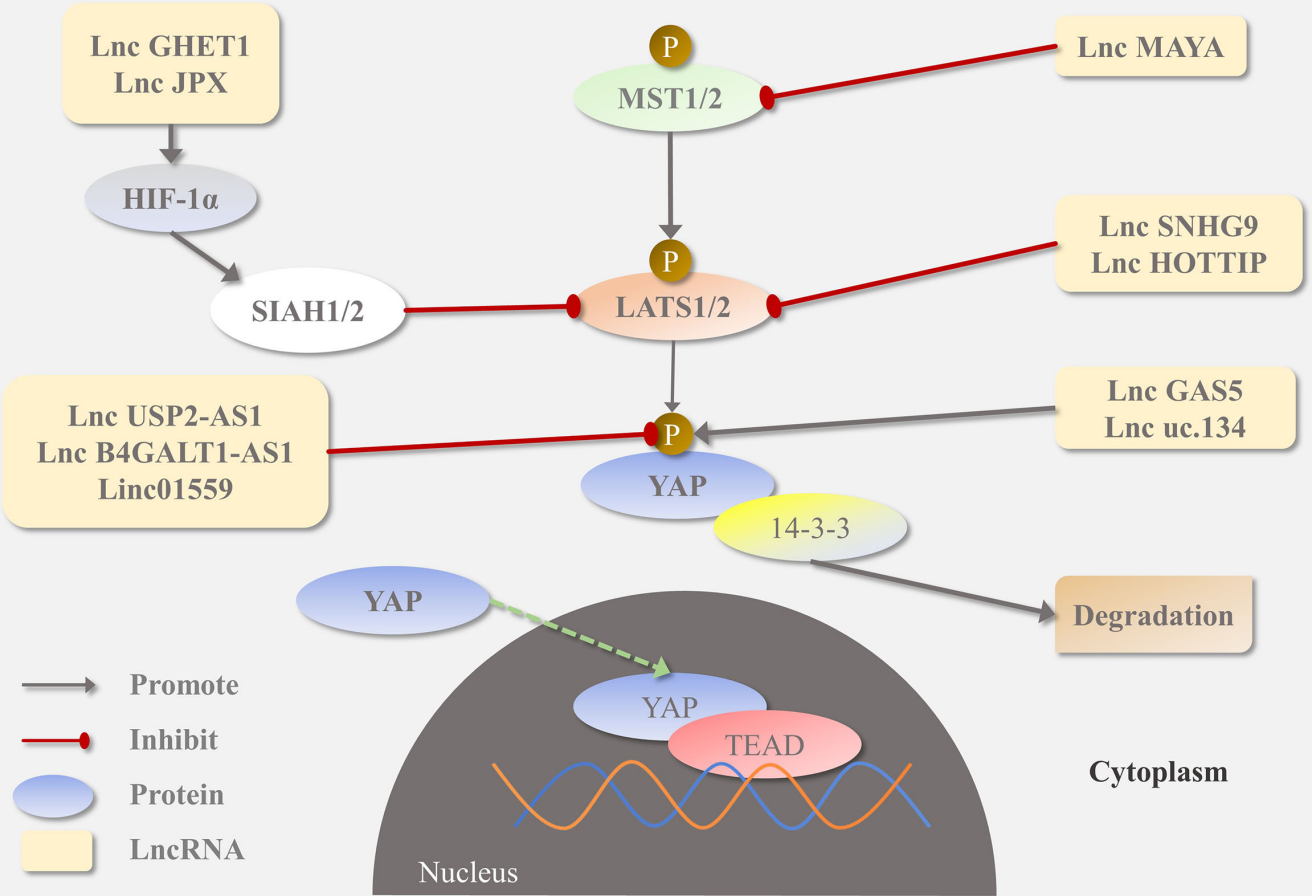
HIPPO-YAP signaling pathway and its regulation by lncRNA.

The Hippo pathway is highly conserved and plays an essential role in organ size regulation, tissue homeostasis, and tumorigenesis (8, 9). In mammals, through activation of the MST-LATS kinase cascade, multiple upstream stimulus signals (e.g., mechanical environment, cellular energy levels, G protein-coupled receptor signals, oxidative stress, and hypoxia) can regulate the localization of YAP, which in turn regulates organ size by controlling cell proliferation and apoptosis. Upon activation, MST1/2 kinase is phosphorylated and forms a complex with SAV1 to phosphorylate LATS1/2, which in turn phosphorylates the transcriptional co-activator YAP in **Figure 2**. This protein is then coupled with 14-3-3 proteins and retained in the cytoplasm or degraded by ubiquitination-dependent proteasomes. Activation of the Hippo pathway inhibits the nuclear YAP import and downregulates the expression of target genes, such as *CTGF* and *CYR61* (10). In contrast, when the Hippo pathway is inhibited, YAP is not phosphorylated and can escape proteasomal degradation and translocate to the nucleus, where it binds to the transcriptional enhanced associated domain (TEAD) family of transcription factors and induces the expression of downstream target genes.

LncRNAs regulate the polarization subtypes of TAMs and affect the proliferation, metastasis, and prognosis of tumors. The Hippo-YAP pathway also affects the TAM polarization. YAP can promote tumor cells to release pro-inflammatory factors and recruit TAMs. In cancer, TAM, YAP, and abnormally expressed lncRNAs can act as molecular markers for diagnosis and prognosis, as well as potential targets for tumor therapy. However, at present, there are not many pieces of literature and experimental studies reported that look at these three as a whole in this context. So, we have logically summarized recent studies, clarified the close association between the three factors and tumorigenesis, and analyzed the outlook of tumor immunotherapy.

## The Close Association Between LncRNA and Tumor Macrophages

### Aberrant Expression of lncRNA in Tumors and TAM

LncRNA is a vital component of cancer immunotherapy (11), regulates gene expression in the form of RNA, participates in cell proliferation, differentiation, tumor angiogenesis, and metastasis, and plays a significant role in the occurrence, development, and metastasis of cancers (12). It is widely believed that lncRNA mediates tumor progression. In non-small cell lung cancer (NSCLC), GNAS-AS1 expression is negatively related to patient overall survival and is significantly enhanced in TAMs from clinical tumor tissues (13). Moreover, prostate cancer-associated transcript 1 (PCAT-1), a newly identified lncRNA, is dysregulated and functions as an oncogene in cancer (14); specifically, high expression of PCAT-1 is correlated with colorectal cancer (CRC) progression (15). As a prostate-specific regulator of cell proliferation, PCAT-1 is a target of the polycomb repressive complex 2 (PRC2) (16). Moreover, in gastric cancer (GC), higher PVT1 expression is significantly associated with greater infiltration depth and advanced TNM stage based on tissues and cell lines, as well as in animal experiments than in normal (17). LncRNA is abnormally regulated in cancer, and in-depth studies on these molecules could help us to better understand and optimize new strategies for tumor treatment.

Macrophages influence tumor metabolism through specific lncRNAs. Researchers found that glycolysis in tumor cells results in the release of lactate, which stimulates the upregulation of hypoxia inducible factor-1α (HIF-1α)-stabilizing long noncoding RNA (HISLA) expression in macrophages, in turn inhibiting the hydroxylation and degradation of HIF-1α by blocking the interaction between PHD2 and HIF-1α, thereby promoting extracellular vesicle (EV) transport and enhancing glycolysis, which results in the production of lactate. Accordingly, this constitutes a feed-forward loop between TAMs and tumor cells. Finally, TAMs facilitate glycolysis and the evasion of apoptosis in breast cancer cells *via* the lncRNA HISLA (18).

### LncRNA Affects Macrophage Recruitment, Polarization, Phenotypic Transition, and Thus, Tumor Immune Escape

LncRNAs can recruit macrophages into tumors to promote metastasis. Studies have generally found that the induction of a macrophage phenotypic switching from M1 to M2 might promote cell proliferation, metastasis, immune evasion, and thus cancer initiation and progression. **Table 1** shows data on the regulation of macrophages by relevant lncRNAs. By directly inhibiting the expression of miR-4319, a miRNA that targets the N-terminal EF-hand calcium-binding protein 3 (NECAB3) to suppress its expression, GNAS-AS1 promotes macrophage M2 polarization and NSCLC cell progression (13). In addition, the overexpression of lncRNA ANCR in macrophages decreases the concentration of IL-1β and IL-6, M1-type macrophage polarization marker molecules, and inhibits macrophage M1 polarization (26). In osteosarcoma, the lncRNA RP11-361F15.2 promotes cytoplasmic polyadenylation element-binding protein 4 (CPEB4)-mediated M2-like polarization of TAMs and tumorigenesis by competitively binding to miR-30c-5p (21).

**Table 1:** LncRNA affects macrophage recruitment, polarization, phenotypic transition.

Positive Role	LncRNA	Tumor	Mechanism	Reference
Macrophages recruitment	LNMAT1	Bladder Cancer	LNMAT1 induces upregulation of CCL2 to recruit macrophages into tumors	(19)
M2 polarization	LncRNA XIST	Lung Cancer	LncRNA XIST downregulation inhibits IL-4-induced M2 polarization	(20)
M2 polarization	GNAS-AS1	NSCLC	GNAS-AS1/miR-4319/NECAB3 axis promotes macrophage M2 polarization	(13)
M2 polarization	RP11-361F15.2	Osteosarcoma	RP11-361F15.2 promotes CPEB4-mediated tumorigenesis and M2-like polarization of TAM through competitive binding to miR-30c-5p	(21)
M2 polarization	RPPH1	Colorectal Cancer	CRC cell-derived exosomes translocate RPPH1 into macrophages and mediate macrophage M2 polarization	(22)
M2 polarization	LINC00662	Hepatocellular carcinoma (HCC)	LINC00662 promotes M2 polarization by inducing the secretion of WNT3A	(23)
Inhibit M2 polarization	CASC2c	GBM	CASC2c binds to FX and inhibits its expression and secretion, which in turn inhibits M2 polarization	(24)
Inhibit M1 polarization	LincRNA-p21	Breast Cancer	LincRNA-p21 knockdown promotes M1 polarization by promoting MDM2 antagonism p53 activation.	(25)
Inhibit M1 polarization	LncRNA ANCR	Gastric Cancer	LncRNA ANCR overexpression inhibits M1 polarization by decreasing IL-1β and IL-6	(26)
M1/M2 phenotype transition	LncRNA cox-2	Hepatocellular carcinoma (HCC)	LncRNA cox-2 siRNA down-regulates IL-12 and TNF-α in M1 and up-regulates IL-10 and Arg-1 in M2 macrophages.	(27)

**Figure 3:**
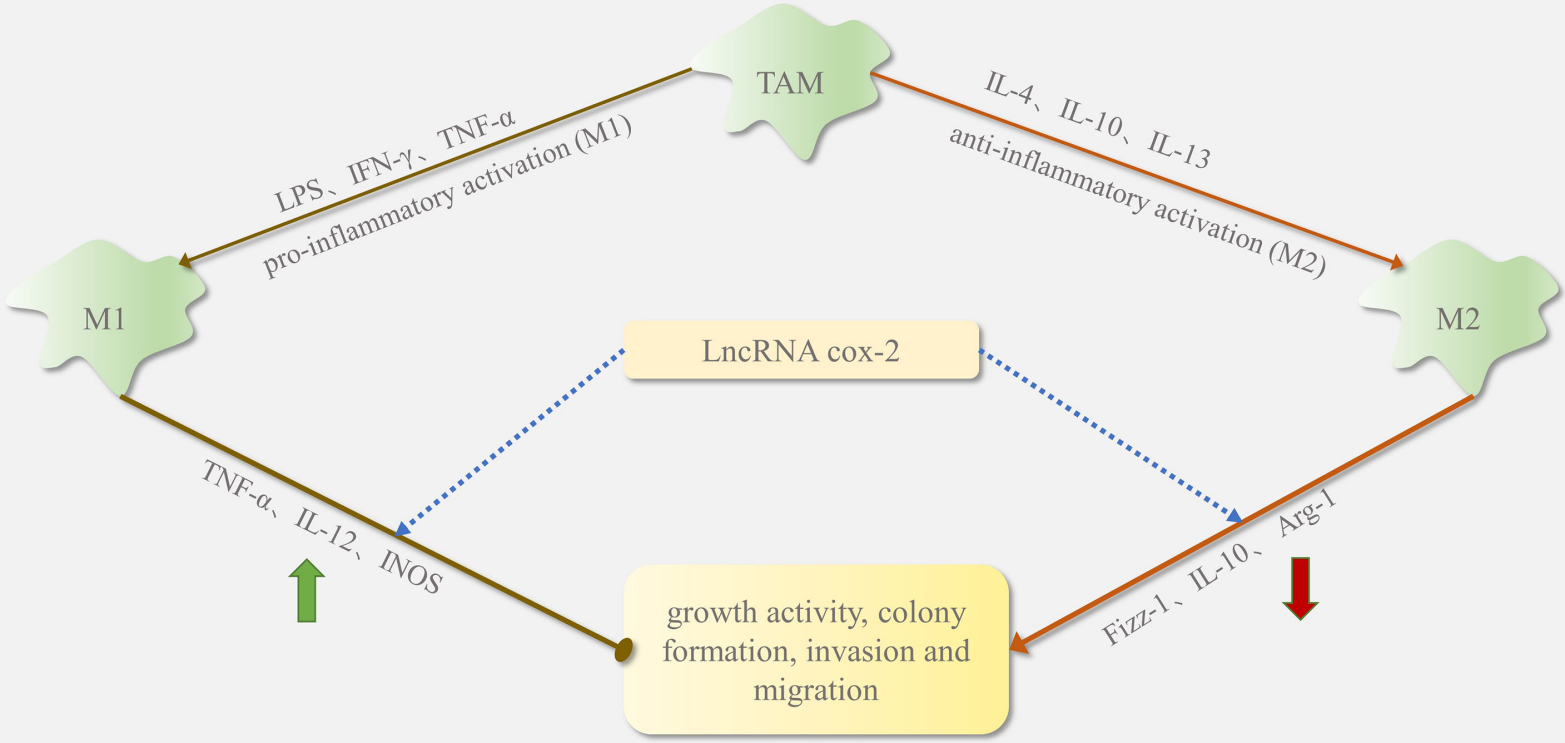
The function of lncRNA cox-2 in TAM phenotype switching.

However, in some cases, lncRNAs can also inhibit M2 cell polarization and macrophage recruitment. LncRNA cox-2 in HCC alters M1/M2 macrophage polarization by regulating the expression of macrophage polarization-related genes (iNOS, TNF-α, Arg-1, and Fizz1). As shown in **Figure 3**, lncRNA cox-2 siRNA decreases IL-12 levels and mRNA expression of TNF-α and iNOS in M1 macrophages and increases IL-10 level and mRNA expression of Arg-1 and Fizz-1 in M2 macrophages (27). By promoting M1 macrophage polarization and inhibiting M2 macrophage polarization, lncRNA cox-2 inhibits the HCC cell proliferation, invasion, migration, angiogenesis, and EMT. Furthermore, silencing the lncRNA Xist in M1 macrophages in breast and ovarian cancers stimulates TAM polarization towards the M2 phenotype and thus proliferation and migration. This suggests that augmented Xist expression in M1 macrophages could be targeted in the treatment of breast and ovarian tumors (2). It can be inferred that the recruitment and polarization of macrophages induced by lncRNA is not a one-sided positive or negative trend.

As expected, lncRNAs are closely correlated with macrophage polarization and tumor progression. Similarly, these macrophage-associated lncRNAs were also cross-linked to the Hippo-YAP pathway. By recruiting heterogeneous nuclear ribonucleoprotein L to the chemokine CCL2 promoter, lymph node metastasis-associated transcript 1 (LNMAT1) activates the CCL2 upregulation, recruits macrophages to the tumor, and promotes lymphatic metastasis *via* vascular endothelial growth factor C (VEGF-C) excretion (19). Coincidentally, researchers have demonstrated that YAP occupies the CCL2 gene and promotes CCL2 expression in mouse cardiac fibroblasts (28). CCL2 is related to increased macrophage infiltration and pro-inflammatory cytokine expression, and YAP expression upregulates the fibrosis and inflammation index. Both LNMAT1 and YAP can promote the expression of CCL2 and then act on macrophages, indirectly affecting inflammation and tumors, suggesting that there may be a closer relationship among lncRNA, YAP, and the macrophages.

Moreover, regarding the induction of M2 polarization, downregulation of lncRNA XIST inhibits interleukin-4 (IL-4)-induced M2 polarization and downregulates the expression of M2-specific markers (e.g., IL-10, Arg-1, and CD163) (20). In the blood and tissue samples of Wilms tumor (WT) patients, researchers found that lncRNA XIST upregulation is correlated with miR-194-5p downregulation and YAP upregulation, suggesting that XIST regulates the miR-194-5p/YAP pathway (29). Furthermore, CRC cell-derived exosomes promote the metastasis and proliferation of CRC cells by translocating lncRNA RPPH1 to macrophages to mediate macrophage M2 polarization. In addition, investigators have found that lncRNA RPPH1 interacts with β-III microtubulin (TUBB3) to inhibit its ubiquitination and induce epithelial-mesenchymal transition (EMT) (22). EMT plasticity plays a critical role in connecting lncRNAs to YAP. As a potent transcriptional coactivator, YAP forms a complex with the zinc-dependent EMT transcription factor ZEB1 to activate integrin α3 (ITGA3) transcription through TEAD binding sites (30). The cancer-promoting zinc transporter ZIP4 promotes EMT plasticity through the ZEB1/YAP1-ITGA3 signaling axis. Through EMT, the indirect connection point, we determined the potential link between lncRNAs and YAP.

Linc00662 promotes hepatocellular carcinoma (HCC) progression and M2 macrophage polarization by upregulating WNT3A expression and secretion through competitive endogenous RNA (ceRNA) mechanism (23). By sponging miR-497-5p, LINC00662 regulates YAP1-mediated GC cell proliferation, and the knockdown of LINC00662 suppresses the Hippo-YAP pathway (31). In addition, as MDM2 (mouse double minute 2) induces proteasome-dependent degradation of p53 and activates the NF-κB and STAT3 pathways, lincRNA-p21 knockdown promotes macrophage polarization to a pro-inflammatory M1 phenotype (25). These observations suggest that lncRNAs inhibit macrophage M1 polarization and promote M2 polarization in the TME, reprogramming their specific functional phenotypes to promote cancer progression and metastasis.

## Complex Interactions Between YAP and LncRNA

The Hippo pathway is a tumor suppressor signaling pathway that restricts its downstream oncogenic effector YAP, as detailed in **Table 2**. In 2021, Lin et al. discovered that lncRNA SNHG9 promotes LATS1 liquid-liquid phase separation and inhibits LATS1-mediated YAP phosphorylation, promoting the oncogenic signaling of YAP. SNHG9 deletion inhibits the growth of xenograft breast tumors (33). Studies have identified crosstalk between the ROR1/HER3-LLGL2-MAYA-NSUN6 signaling axis and the Hippo-YAP pathway, with the former methylating the MST1 site to eliminate MST1 kinase activity and activate YAP and its target genes to promote bone metastasis (32). Another study illustrated that LINC01559, lncRNA B4GALT1-AS1, and lncRNA USP2-AS1, a Yes-associated protein 1 (YAP1)-binding lncRNA, can interact with YAP proteins, hinder YAP phosphorylation, recruit YAP to the nucleus, and trigger the expression of downstream target genes to accelerate tumor progression (35–37). Interestingly, in OS stem cells, B4GALT1-AS1 recruits HuR to enhance the stability of *YAP* mRNA and its transcriptional activity, thereby promoting OS cell stemness and migration (38). In addition, lncRNA can upregulate YAP levels in other ways. 

**Table 2:** Regulation of HIPPO-YAP pathway by lncRNA.

Role	Target molecules	LncRNA	Disease	Role	Reference
Contribution to YAP	MST	LncRNA MAYA	Bone metastasis	The ROR1/HER3-LLGL2-MAYA-NSUN6 signaling axis methylates the MST1 site and eliminates MST1 kinase activity	(32)
Contribution to YAP	LATS	LncRNA SNHG9	Breast Cancer	LncRNA SNHG9 promotes LATS1 liquid-liquid phase separation for oncogenic YAP	(33)
Contribution to YAP	LATS	HOTTIP	Osteosarcoma (OS)	HOTTIP catalyzes LATS2 promoter methylation	(34)
Contribution to YAP	YAP	LINC01559	Pancreatic cancer	LINC01559 interacts with YAP protein and blocks YAP phosphorylation	(35)
Contribution to YAP	YAP	lncRNA USP2-AS1	Colon Adenocarcinoma (COAD)	lncRNA USP2-AS1 binds YAP1 and decreases p-YAP (S127)	(36)
Contribution to YAP	YAP	B4GALT1-AS1	Osteosarcoma (OS)	B4GALT1-AS1 recruits HuR to enhance the stability of YAP mRNA	(37)
Contribution to YAP	YAP	B4GALT1-AS1	Colon Cancer	B4GALT1-AS1 binds to YAP and recruits YAP to the nucleus	(38)
Contribution to YAP	YAP	LncRNA-ATB	Hepatocellular carcinoma (HCC)	LncRNA-ATB reduces p-YAP expression and induces YAP nuclear translocation	(39)
Contribution to YAP	YAP	METTL3	NSCLC	METTL3 promotes YAP mRNA translation and increases YAP mRNA stability by regulating the MALAT1-miR-1914-3p-YAP axis	(40)
Contribution to YAP	YAP	MALAT1	Diabetic cardiomyopathy (DCM)	MALAT1 positively regulates the nuclear translocation of YAP by binding to CREB	(41)
Contribution to YAP	HIF-1α	LncRNA JPX	Intervertebral disc degeneration (IDD)	Overexpression of lncRNA JPX suppresses miR-18a-5p, upregulates HIF-1α and thereby inhibits Hippo-YAP pathway	(42)
Contribution to YAP	HIF-1α	LncRNA GHET1	Triple-negative breast cancer (TNBC)	LncRNA GHET1 knockdown reduces the level of HIF-1α phosphorylation to retain YAP in the cytoplasm.	(43)
Suppression of YAP	YAP	Lnc-mi497RHG	Bladder Cancer	Lnc-mi497RHG synergistically inhibits Hippo-Yap and TGF-β pathways, especially attenuating the interaction between Yap and Smad3	(44)
Suppression of YAP	YAP	GAS5	Colorectal Cancer	GAS5 directly interacts with the YAP WW structural domain, promoting YAP phosphorylation and ubiquitin-mediated degradation	(45)
Suppression of YAP	LATS	LncRNA uc.134	Hepatocellular carcinoma (HCC)	LncRNA uc.134 inhibits CUL4A-mediated LATS1 ubiquitination and increases YAPS127 phosphorylation	(46)

However, lncRNA can also inhibit tumor cell viability and growth through YAP inactivation. The miRNA–host gene lncRNA (lnc–miRHG) association produces miRNAs and regulates cancer progression. MiR-497 and miR-195, derived from MIR497HG, synergistically inhibit Hippo/Yap and transform growth factor β (TGF-β) signaling, thereby attenuating the interaction between YAP and Smad3 (44). CUL4A is a scaffolding protein of the ubiquitin-proteasome system and a ubiquitin E3 ligase that mediates LATS1 ubiquitination (53). A novel lncRNA, uc.134, inhibits HCC progression by suppressing CUL4A expression and increasing YAPS127 phosphorylation (46). Furthermore, talazoparib, a novel and potent poly(ADP-ribose)polymerase-1/2 (PARP1/2) inhibitor, induces the lncRNA PLK4, which inhibits the viability and growth of HCC cells through YAP inactivation and cellular senescence, thus acting as an oncogene suppressor (54). To summarize, lncRNAs can positively or negatively regulate YAP levels and directly or indirectly affect tumor progression.

Not only can lncRNAs affect YAP expression levels, but YAP can also interfere with the Hippo-YAP pathway to indirectly act on tumors; and the two can even interact with each other. The multiple repeat sequences of the lncRNA NORAD bind to and segregate S100P, and the S100P decoy function inhibits the migration, invasion, and metastasis in lung and breast cancers. Transduction of the Hippo pathway YAP/TAZ-TEAD complex transcriptionally represses the NORAD expression, together with the action of the NuRD complex (47). Furthermore, researchers have found that the YAP/TEAD1 complex and lncRNA both influence each other to form a feed-forward circuit. As a ceRNA of miR-484, THAP9-AS1 attenuates the inhibitory effect of miR-484 on YAP, leading to the YAP upregulation. Moreover, THAP9-AS1 binds to YAP protein and inhibits LATS1 phosphorylation. Notably, the YAP/TEAD1 complex promotes THAP9-AS1 transcription, forming a feed-forward circuit (55). The regulatory relationship between lncRNAs and the Hippo-YAP pathway is complex and variable, and there is still much scope for research and exploration. Further, a better understanding of the mechanisms underlying their roles in tumors is of strategic importance for future immune-targeted therapies.

As mentioned above, some lncRNAs can affect the HIPPO-YAP pathway, and lncRNAs and YAP interact with each other. However, these alone are not sufficient to help us understand the relationship between the two. We were surprised to find that some lncRNAs regulating the HIPPO-YAP pathway could be directly or indirectly linked to macrophages. The Hippo pathway YAP/TAZ-TEAD complex transcriptionally represses lncRNA NORAD expression. NORAD could serve as a diagnostic marker for neonatal sepsis (NS) patients. NORAD knockdown reversed the overexpression of IL-6, IL-8, and TNF-α pro-inflammatory cytokines in macrophages under LPS conditions (48). The lncRNA GAS5 interacts directly with the WW structural domain of YAP, facilitating YAP phosphorylation and subsequent ubiquitination-mediated degradation, thereby inhibiting CRC progression (45). lncRNA knockdown was performed in M2 macrophages, and the effect on polarization was assessed by surface marker analysis. Knockdown of GAS5 results in the downregulation of M2 surface markers (CD163 and CD206) and a concomitant increase in M1 markers (MHC II or CD23), which highlights the instrumental role of lncRNA GAS5-mediated regulation of macrophage differentiation and polarization (50). Similarly, in cells isolated from diabetic (Db) wounds, lncRNA GAS5 is dysregulated, and GAS5 loss-of-function may be partly responsible for the persistence of M1 macrophages and enhancement of Db wound healing (51).

In addition to participating in the regulation of the Hippo-YAP pathway, these lncRNAs are directly related to the M1/M2 polarization of macrophages. However, some lncRNAs are indirectly associated with macrophages. The lncRNA HOTTIP (human homeobox A transcript) in osteosarcoma (OS) catalyzes LATS2 promoter methylation, inhibits LATS2 expression, activates YAP, initiates downstream target gene expression, and maintains OS cell viability, proliferation, migration, and invasion (34). Meanwhile, researchers found that lncRNA HOTTIP can decoy miR-27a-3p to promote G protein subunit gamma 12 (GNG12)-mediated metastasis in osteosarcoma. GNG12 suppresses adaptive immunity to influence the tumor microenvironment by inhibiting M1 and M2 macrophage infiltration (49). Similarly, in triple-negative breast cancer (TNBC), hypoxia induces lncRNA GHET1, and lncRNA GHET1 knockdown reduces HIF-1α phosphorylation under hypoxic conditions, retaining YAP in the cytoplasm. LncRNA GHET1 overexpression promotes nuclear translocation of YAP and TNBC progression (43). In adipose-derived mesenchymal stem cells (ASCs), researchers demonstrated that ASCs-derived interleukin 10 (IL-10), mediated by HIF-1α, plays a crucial role in enhancing macrophage recruitment and inducing macrophages toward the M2 phenotype (52). **Table 3** shows more systematic information.

**Table 3:** Direct or indirect crosstalk among YAP, LncRNA, and TAM.

LncRNA	Relate to Hippo-YAP	Relate to TAM	Reference
LNMAT1	YAP occupies the CCL2 gene and promotes CCL2 expression	LNMAT1 induces upregulation of CCL2 to recruit macrophages into tumors	(19, 28)
LncRNA XIST	LncRNA XIST upregulation is correlated with YAP upregulation	LncRNA XIST downregulation inhibits IL-4-induced M2 polarization	(20, 29)
RPPH1	LncRNA RPPH1 and YAP are linked indirectly by EMT	CRC cell-derived exosomes translocate RPPH1 into macrophages and mediate macrophage M2 polarization	(22, 30)
LINC00662	The knockdown of LINC00662 suppresses the Hippo-YAP pathway	LINC00662 promotes M2 polarization by inducing the secretion of WNT3A	(23, 31)
LncRNA NORAD	Hippo pathway YAP/TAZ-TEAD complex transcriptionally represses lncRNA NORAD expression	NORAD knockdown reversed the overexpression of IL-6, IL-8, and TNF-α pro-inflammatory cytokines in the macrophage cells	(47, 48)
HOTTIP	HOTTIP catalyzes LATS2 promoter methylation	LncRNA HOTTIP promotes GNG12 expression, which inhibits M1 and M2 macrophage infiltration	(34, 49)
GAS5	GAS5 directly interacts with the YAP WW structural domain, promoting YAP phosphorylation and ubiquitin-mediated degradation	Knockdown of GAS5 shows downregulation of M2 surface markers and concomitant increase in M1 markers	(45, 50, 51)
LncRNA GHET1	LncRNA GHET1 knockdown reduces the level of HIF-1α phosphorylation to retain YAP in the cytoplasm.	HIF-1α enhances macrophages recruitment and inducing macrophages toward M2 phenotype	(43, 52)

## The Close Relationship Between YAP and Macrophages

### YAP Stimulates Macrophage Production of Proinflammatory Cytokine Factors and Inflammatory Responses

Various studies have demonstrated that YAP regulates the inflammatory response of macrophages through various pathways. Regarding the mechanism through which the Hippo-YAP pathway regulates macrophage behavior, it was found that the overexpression of YAP exacerbated the titanium ion-induced NF-κB pathway-mediated inflammatory response. Titanium ions induce YAP expression and activate the NF-κB pathway to upregulate proinflammatory cytokine expression in macrophages (56). By inducing the interaction between YAP and NF-κB subunit p65, LPS stimulates NF-κB activation and TNFα production in macrophages, but this process is dependent on YAP activation and nuclear translocation (57). Lactate significantly inhibited macrophage NF-κB and YAP activation and nuclear translocation owing to YAP inactivation, which is mediated by GPR81-dependent AMKP and LATS activation-mediated YAP phosphorylation. Lactate reduces the interaction between YAP and NF-κB, thereby inhibiting the production of TNF-α and IL-6. Lactate inhibits YAP and NF-κB activation *via* GPR81-mediated signaling and suppresses the inflammatory response of macrophages following LPS stimulation. In addition, activator protein 1 in macrophages/Kupffer cells (KCs) promotes the LPS transcriptional activation of YAP. This further enhances the expression of proinflammatory cytokines, including monocyte chemotactic protein 1, tumor necrosis factor α, and interleukin 6, through binding to the TEA domain in the promoter regions of genes encoding inflammatory factors. YAP in KCs enhances the production of proinflammatory factors and causes nonalcoholic steatohepatitis (58). YAP/TAZ was found to increase IL-6 expression to promote the proinflammatory response and interact with the NCoR1 inhibitor complex to decrease arginase-i (Arg1) expression and inhibit the reparative response (59). The initial proinflammatory response, followed by an anti-inflammatory response, is critical for reducing myocardial infarction injury and promoting healing and scar formation. YAP/TAZ deficiency impairs the early inflammatory response and promotes the timely polarization of macrophages from the proinflammatory to reactive phenotype. Furthermore, the cellular adhesion microenvironment regulates the macrophage inflammatory response through YAP. Soft matrix reduces the expression of inflammatory cytokines and YAP in macrophages (60). The identification of YAP as a key molecule in the control of macrophage inflammation has broad implications for the regulation of macrophages in health and disease.

### YAP Activation Is the Basis for Macrophage Recruitment

Factors, such as colony-stimulating factor 1 (CSF1) and CCL2, secreted by the TME recruit macrophages to the tumor, and activation of the effector YAP in the Hippo pathway underlies the recruitment of macrophages by tumor-initiating cells (TICs). It has been demonstrated that TICs recruit M2 macrophages at the monocytic stage and that the YAP-TEAD transcriptional complex directly or indirectly activates the transcription of growth factor CSF1 and chemokine CCL2, respectively, thereby promoting TIC survival and tumorigenesis (61). When studying mouse hepatocytes, it was found that gene deletion of *Mst1*/*Mst2* upregulated monocyte chemoattractant protein-1 (*Mcp-1*) expression, mixed M1 and M2 phenotypic of macrophage infiltration, and promoted HCC development. Removal of YAP eliminates abnormal *Mcp-1* expression and restores normal liver growth in mice (62). This study identified MCP1 as a direct transcriptional target of YAP. Moreover, Hippo signaling in hepatocytes inhibits Yap-dependent *Mcp-1* expression, which in turn inhibits macrophage infiltration and thus maintains normal liver growth. Similarly, elevated YAP levels have been shown to exacerbate macrophage infiltration and MCP-1 expression in other studies such as with liver injury (63), acute kidney injury-chronic kidney disease (AKI-CKD) transition (64), and atherosclerosis (65). These studies highlight the critical clinical significance of Hippo signaling in suppressing the inflammatory and TME. Hippo signaling inactivation and YAP activation induce MCP-1-mediated macrophage infiltration and tumor development, suggesting that more effective therapeutic interventions could be employed in the future to refine targeted therapies.

### YAP Activation and M1/M2 Macrophage Polarization

High YAP expression in tumors can result in macrophage polarization to an M2-like phenotype. TNBC cells upregulate YAP expression in macrophages, which induces macrophage polarization to an M2-like phenotype (66). In addition, augmented YAP activation in M2 macrophages promotes TNBC metastasis *via* the MCP-1/CCR2 pathway. Nogo-B, an endoplasmic reticulum-resident protein, also known as reticulon 4 B, promotes HCC progression by enhancing Yap-mediated TAM M2 polarization, a process that is blocked by the YAP inhibitor verteporfin (67). A similar phenomenon has been observed in renal fibrosis. The Wnt5a signaling protein enhances TGFβ1-induced macrophage M2 polarization and YAP transcriptional co-activator expression (68). Verteporfin also blocks TGFβ1- and Wnt5a-induced macrophage M2 polarization. In aortic dissection, angiotensin type 1 receptor (AT1R) binding to Ang II induces YAP phosphorylation and further promotes macrophage polarization toward an M1 phenotype with endothelial cell adhesion (69). In addition, researchers found that YAP1 overexpression indirectly enhances drug resistance in tumor cells. YAP overexpression in GC cells induces M2 polarization in macrophages, which secrete CCL8 and activate phosphorylation of the JAK1/STAT3 signaling pathway components, thereby enhancing 5-FU resistance in tumor cells (70). Therefore, targeting YAP to overcome chemoresistance and tumor immunotherapy is a potential approach in the future.

However, the induction of macrophage polarization by YAP was not the only possibility for M2 macrophages. Mechanical ventilation causes lung injury and inflammation and upregulates YAP expression in lung macrophages. YAP deficiency in macrophages attenuates lung injury and reduces the production of proinflammatory cytokines, such as IL-1β and TNF-α. YAP deficiency enhances M2 polarization while inhibiting M1 polarization (71). The M2 macrophage-derived exosome miR-590-3p targets LATS1 and subsequently activates YAP/β-catenin transcription in mouse colonic epithelial cells to reduce inflammation and promote epithelial regeneration, attenuate DSS-induced mucosal damage, and promote epithelial repair (72). Interestingly, however, another study found that YAP in macrophages exacerbates inflammatory bowel disease (IBD); however, YAP promotes epithelial cell regeneration, which enhances IBD recovery (73). M1 macrophages and proinflammatory cytokines exacerbate IBD symptoms, whereas M2 macrophages promote tissue repair, attenuate inflammation, and alleviate IBD. YAP blocks IL-4/IL-13-induced M2 polarization, while promoting LPS/IFN-γ-triggered M1 macrophage activation. In summary, we found different scenarios in which YAP inhibits M1 or M2 macrophage polarization. Therefore, we should consider the possibility that targeting YAP to inhibit tumor growth might promote tumor growth by activating TAMs. In other words, this factor exerts different effects in various cell types, like increasing tumor proliferation and metastasis, regulating M2/M1 macrophage polarization, promoting epithelial regeneration, and producing inflammatory responses, among others. Therefore, therapeutic approaches targeting YAP should consider the appropriate cell type.

YAP affects macrophage recruitment, polarization, and production of pro-inflammatory factors. However, it is currently unknown whether lncRNAs are involved in the mechanism of macrophage polarization *via* the Hippo pathway. Researchers are more likely to look at the effects of each of the three on tumors independently or at the interconnections between the two. In this study, the three are seldom combined as a whole to carry out in-depth analysis and experiments. Studies in this direction are insufficient, and more attention should be paid to the future. In the occurrence and development of tumors, it is of great significance to study the independent effects and interaction mechanisms of lncRNA, YAP, and TAM, three important therapeutic targets, to understand the initiation, metastasis, treatment, and prognosis of cancer. Only in this way can we better advance and expand cancer therapies based on the three conventional and non-conventional therapies.

## Discussion

As potential targets in recent years, lncRNAs, YAP, and TAMs have been associated with tumor development, proliferation, and metastasis. It was found that lncRNA, as a potential target, can affect tumor immunity. LncRNA GAS5 expression is decreased in NK cells from HCC patients, and the downregulation of GAS5 expression inhibits the cytotoxicity of NK cells. Overexpression of GAS5 increases the secretion of interferon-c (IFN-c) and enhances the cytotoxicity of NK cells (74). Moreover, indoleamine 2,3-dioxygenase (IDO) induces the differentiation and maturation of T regulatory cells (Tregs) to suppress T-cell immunity. Interfering with lncRNA SNHG1 promotes the elevation of miR-448 levels, decreases IDO levels, and thus inhibits the differentiation of Treg cells and attenuates immune escape in breast cancer (75). However, lncRNAs also promote immunosuppression and interfere with the clearance of tumors by the immune system. LncRNA epidermal growth factor receptor (EGFR) stimulates Treg differentiation, inhibits cytotoxic T lymphocyte (CTL) activity, and promotes HCC growth through EGFR (76). LncRNA NKILA inhibits NF-κB activity, allowing the immune-mediated clearance of activated T lymphocytes to promote tumor immune evasion. Meanwhile, investigators demonstrated that targeting lncRNA in T cells from secondary metastasis tumors is feasible, greatly regulating lncRNA expression (77). In addition, the expression of target-lncRNAs in metastatic cells can be modified by using gene modification techniques, such as CRISPR-Cas9 or small interfering RNA (77).

For macrophages, however, the antitumor activity in malignant cancers has the potential to act as a therapeutic target. A cluster of differentiation 47 (CD47) is widely overexpressed in various malignancies and might be a predictor of poor prognosis and tumor metastasis (78). CD47 inhibits macrophage phagocytosis in ovarian cancer cells, and its downregulation or inhibition enhances the antitumor effect of macrophages (79). Similarly, in malignant melanoma, activation of killer macrophages, either through the *in vitro* activation of macrophages *via* transmigration or *in vivo* activation of macrophages, in combination with other treatments, such as surgery, chemotherapy, and radiotherapy, might provide an effective and comprehensive strategy for targeting the aggressive metastatic capacity and therapy resistance of melanomas (80). However, another therapeutic strategy is to target TAMs in the TME. CSF-1 allows macrophages to differentiate and survive. Researchers used CSF-1 receptor (CSF-1R) inhibitors to target TAMs in a mouse GBM model, which significantly improved mouse survival and tumor regression (81). Unfortunately, targeted TAM treatment strategies are not as effective. To date, immune interventions for GBM patients have not been successful because of the presence of a TME that promotes tumor growth and immune escape. Recently, researchers have developed novel platforms for evaluating genetically engineered macrophages (GEMs) (82). GEMs resist reprogramming mediated by tumor secretory factor signaling, override the immunosuppressive effects of the TME, and support existing or new immunotherapies.

The effectiveness of immunotherapeutic strategies against TAMs is limited (83); therefore, considering the significant role of the Hippo-YAP pathway in TAMs, investigators have identified YAP as a potential target for tumor-targeted therapy. Recent studies have shown a binary classification of cancers into YAP-on and YAP-off based on the presence or absence of functioning YAP proteins. These two cancer states switch to each other, leading to the development of drug resistance. Yap-off/Yap-on exhibits a unique vulnerability that facilitates the choice of treatment options. Thus, the development of tumor cell resistance can be inhibited by blocking this YAP state transition, which provides a new therapeutic strategy for tumor types with strong aggressive properties (84). In addition, YAP1 inhibition can suppress the recruitment of myeloid-derived suppressor cells (MDSCs), activate effector T-cell activity, and enhance sensitivity to immune-oncologic agents (IO) drugs, thereby modulating immunosuppression of the TME. YAP1 inhibition in combination with anticancer drug therapy might be a promising therapeutic strategy (85). In addition, many investigators have gradually discovered the therapeutic value of single-target therapies in combination with immunotherapy. A recent study showed that focal adhesion kinase (FAK), a potential therapeutic target upregulated in tumors of intrahepatic cholangiocarcinoma (iCCA), promotes tumorigenesis in mice by inducing YAP (86). The oncogenic potential of FAK was investigated in conditional FAK-knockout mice and inducible Cre mice, and the potential to target FAK for iCCA was studied based on *in vitro* and *in vivo* drug treatments. Activation of the CDK4/6 pathway in mouse and human iCCA suggests that combined targeting with anti-CDK4/6 inhibitors could be an effective treatment strategy. Notably, the HIPPO/YAP pathway is severely dysregulated in alcoholic hepatitis (AH), with uncontrolled activation of YAP leading to hepatocyte transdifferentiation to the biliary phenotype and the loss of hepatocyte identity with impaired regeneration (87). Using animal models, experimental cells, and human samples of AH and alcoholic cirrhosis, investigators conjunctively found that the reversal of hepatocyte defects mediated by YAP inhibition appears to be a therapeutic strategy for AH regenerative treatment. This is in contrast to the effects of YAP, which has been shown to promote early hepatocyte cycle progression. This suggests that the effects of transient and sustained YAP activation could be quite different, and for tumors, researchers also need to be aware of the possible different effects of the differential timings of YAP activation.

In addition, researchers are increasingly aware that the tumor immune microenvironment is multi-layered and complex, and we should not target cancer in isolation but consider it as part of the TME ecosystem. Although research on the complex composition and activity of the TME is relatively superficial, the TME is more heterogeneous across species or stages of progression, and disrupts the tumor ecosystem could be achieved by targeting multiple heterogeneous cell populations in the tumor and microenvironment (88). In summary, there are several possible directions for future immunotherapeutic strategies for tumors as follows: one is to improve the body’s immune capacity, promote immune activation, such as with macrophages and NK cells to kill tumors, and influence the immune status of the TME; second, to combine multiple targets, such as through the use of traditional radiotherapy modalities and immunotherapy, or multiple different immunotherapies acting in combination, and the detection of various novel markers to identify potentially effective drug targets, such as lncRNA and YAP, which are closely related to tumors. Likewise, this requires a deeper understanding of the TME and its internal components with respect to the mechanisms of tumorigenesis and immunotherapy.

However, there are few studies on whether lncRNAs affect macrophages by regulating the Hippo-YAP pathway, or whether lncRNAs are involved in the mechanism of macrophage polarization *via* the Hippo-YAP pathway. More studies have focused on the mechanism by which YAP regulates macrophage polarization or lncRNAs regulating macrophage polarization or phenotypic transformation to promote tumor proliferation and metastasis. Perhaps in the research direction of studying lncRNA, TAM, and YAP, researchers can try to broaden their thinking and connect the three to build a network instead of being limited to the most common and classic research approaches. More in-depth and specific studies are needed to determine whether the relationship among the three is in the linear lncRNA-YAP-TAM axis, the interconnected loop, or a more complex network relationship. Revealing the key role of lncRNA, YAP, and TAM linkages in tumorigenesis and development can provide different ideas for tumor treatment, expand the targets of traditional therapy and immunotherapy, improve the prognosis of clinical patients, and reduce mortality, which has extremely important practical significance. Moreover, given that tumorigenesis and development is a dynamic process, the metabolic patterns of cells vary greatly in the early and late stages. At an early stage, glycogen accumulation and phase separation in liver tumors can activate YAP to drive liver tumor initiation. However, in advanced stages, YAP can be activated by a different mechanism, high levels of glucose, to promote tumor development (89). It is worth considering whether lncRNAs and TAMs are involved in such completely different YAP activation mechanisms in the early and late stages. Considering the previously identified tumor mechanisms in a new way in the context of YAP, TAM, and lncRNA, crosstalk may lead to new and meaningful findings.

## Conclusion

In summary, lncRNAs, YAP, and TAMs are closely related, and all three are associated with tumor development, proliferation, and metastasis. They have also become potential targets for tumor-targeted therapy in recent years. In the future, we should gain a deeper understanding of the mutual regulation of lncRNA, YAP, and TAMs in the TME and systematically investigate their synergistic tumorigenic mechanism. This has key implications for the combination of antitumor and multi-target immunotherapeutic agents. It would also be worthwhile to investigate how to override the immunosuppressive effects of the TME and avoid the adaptive drug resistance caused by increased YAP expression and M2 macrophages, thus significantly improving the prognosis and survival of cancer patients. Therefore, an in-depth study of the response mechanisms of lncRNA, YAP, and TAMs in the unique ecosystem of the TME could help to address the phenomenon of suboptimal therapeutics in tumor immunotherapy and provide a basis for the discovery of new therapeutic targets.

## Author Contributions

JX, X-YLiu, and QZ reviewed literature and originally drafted the manuscript. HL, PZ, Z-BT, and C-PZ contributed to edit and embellished the manuscript. JX approved the final version of the manuscript. All authors contributed to the article and approved the submitted version.

## Funding

The study was supported by the National Natural Science Foundation (No. 81802777), the “Clinical medicine + X” scientific research project of Affiliated Hospital of Qingdao University, and Qingdao Chinese Medicine Technology Project (2021-zyym26).

## Conflict of Interest

The authors declare that the research was conducted in the absence of any commercial or financial relationships that could be construed as a potential conflict of interest.

## Publisher’s Note

All claims expressed in this article are solely those of the authors and do not necessarily represent those of their affiliated organizations, or those of the publisher, the editors and the reviewers. Any product that may be evaluated in this article, or claim that may be made by its manufacturer, is not guaranteed or endorsed by the publisher.
